# Mortality related factors on hypoxic ischemic encephalopathic patients treated with therapeutic hypothermia: an 11-year single-center experience

**DOI:** 10.55730/1300-0144.5375

**Published:** 2022-03-13

**Authors:** Mehmet Fatih DEVECİ, Hatice TURGUT, Meral ALAGÖZ, Hüseyin KAYA, İsmail Kürşad GÖKÇE, Ramazan ÖZDEMİR

**Affiliations:** Mehmet Fatih Deveci, MD; Division of Neonatology, Department of Pediatrics, Inonu University School of Medicine, Turgut Özal Medical Center, Malatya, Turkey

**Keywords:** Hypoxic ischemic encephalopathy, neonatal encephalopathy, perinatal asphyxia, therapeutic hypothermia

## Abstract

**Background/aim:**

Hypoxic-ischemic encephalopathy (HIE) is a condition that may cause multiple organ dysfunction and has a high rate of mortality and morbidity. Therapeutic hypothermia is the only proven treatment that decreases the sequel and mortality rate of neonates that are born after 36 weeks of pregnancy and have moderate-severe HIE.

**Material and method:**

Our study was a single-center, retrospective study that includes newborns (gestational age ≥ 36 weeks) who underwent therapeutic hypothermia due to hypoxic-ischemic encephalopathy between 2010 and 2020. We evaluated 125 patients who were diagnosed with moderate to severe HIE and received therapeutic hypothermia. Demographic and clinical data were obtained from electronic medical records and patient files. The patients were separated into two groups as exitus group (n = 39) and discharged group (n = 86). We aimed to evaluate factors affecting mortality.

**Results:**

We determined that the median resuscitation times were longer in the delivery room [retrospectively, 10^th^ minutes (0–30) vs. 1 min (0–20), p < 0.05], the tenth min APGAR scores were lower [respectively, 4 (0–7) vs. 6 (3–10), p < 0.05], and the median pH value in the first blood gas taken was lower [respectively, 6.87 (6.4–7.14) vs. 6.90 (6.58–7.12), p < 0.05] in the exitus group. We also determined that multiple organ dysfunction is seen more often in the exitus group.

**Conclusion:**

This study demonstrated that the depth of acidosis in the blood gas, multiple organ dysfunction, and the existence of early-onset seizures are the signs of poor prognosis. Therefore, physicians need to be aware of such prognostic factors to follow these patients more closely in terms of possible complications and to inform their parents.

## 1. Introduction

Hypoxic-ischemic encephalopathy (HIE) is an acute and possibly progressive condition that emerges as a result of intrapartum or postpartum hypoxia and ischemia. Due to insufficient gas exchange across the placenta in the intrauterine period or insufficient pulmonary ventilation in the postnatal period, oxygen and carbon dioxide exchange is disrupted and the patients develop hypoxemia, hypercarbia, and acidosis [1,2]. The incidence rate of HIE is 2–9 per thousand live births [3]. According to the 2008 data of the Turkish Neonatology Association, the rate of HIE in Turkey is 0.26% [4]. HIE is still a significant cause of mortality in neonates. In survivors, it may leave significant neurologic sequels such as vision and/or hearing loss, epilepsy, mental retardation, and cerebral palsy [1,5]. To reduce to mortality and sequel rates of neonates that are experienced HIE, neuroprotective treatments should be started as soon as possible. Nowadays, therapeutic hypothermia is the most trustworthy and strong option which effectiveness-proven in reducing mortality and morbidity among neuroprotective treatments [6]. A meta-analysis of 11 randomized controlled trials published in 2013 found that hypothermia treatment reduced death and neurodevelopmental impairment in 1505 infants with HIE [7].

In this study, we aimed to evaluate the factors related to mortality of moderate-severe HIE patients who were applied therapeutic hypothermia in our neonate intensive care unit within 11 years.

## 2. Material and method

### 2.1. Patient selection

This study had ethical approval from Faculty of Medicine of İnönü University, Malatya, Turkey, and was designed as a single-center, retrospective cohort study.

The neonates who were diagnosed with moderate-severe HIE, hospitalized in our intensive care unit, and received therapeutic hypothermia treatment between the dates of January 2010 and December 2020 were included in our study. Electronic medical records and patient files were reviewed retrospectively. Data taken from the patients’ folder retrospectively were as follows: demographic information (birth weight, sex, age of pregnancy, delivery method and place, maternal age), laboratory examinations (blood gas, kidney and liver function tests, complete blood count, and the electrolyte levels), seizure history, need for intubation, amplitude-integrated electroencephalogram (aEEG) monitoring and cranial ultrasonography findings. Patients were separated into two groups as exitus and discharged ones. To determine the factors affecting mortality, a series of analyses were carried out between the groups.

### 2.2. Management and protocol of therapeutic hypothermia

In our unit, the Tecotherm Neo device (Inspiration Healthcare, UK) is used to induce therapeutic hypothermia. During the treatment, patients are being monitored. The core temperature of the patients is monitored with a rectal temperature probe and stabilized between 33.5 °C–34.5 °C for the entire 72 h. Vital findings and rectal temperature values of the patients are hourly recorded. Urine output, blood gas, and kidney-liver function tests of the patients are closely followed. Blood tests are taken within the first 24 h and repeated later when necessary.

Hypothermia treatment indications in our unit are as follows [4, 8, 9]: 1) The neonates who are in the first postnatal 6 h and age of pregnancy is ≥ 36 weeks; 2) Cord or blood gas which is taken in the first hour of after birth: pH value < 7.00 or base excess (BE) < −16 mmol/L; 3) 10^th^ min APGAR score < 5 or continued resuscitation need; 4) Moderate-severe HIE diagnosis in clinical evaluation according to Sarnat and Sarnat staging; 5) The existence of moderate-to-severe trace irregularity on aEEG if used; 6) Accompanied by low APGAR or encephalopathy findings in patients with pH value < 7.00–7.15 and BE −10–(−16).

### 2.3. Statistical analysis

SPSS for Windows version 21.0 packaged software was used for statistical analysis. Shapiro Wilk test was used in the eligibility check of continuous variables to normal distribution. Independent student t-test was used in the normally distributed variables’ 2 independent groups comparison. Mann Whitney U test was used in the nonnormally distributed variables’ 2 independent groups comparison. While normal continuous data were stated as average ± standard deviation; nonnormal continuous data were stated as median (minimum-maximum). Chi-squared and Fischer exact analysis were used in the test of correlation among categorical variables. P < 0.05 was accepted as significant statistically.

## 3. Results

Between our study’s dates, 14,459 patients were hospitalized in our neonate intensive care unit. One hundred and twenty-five patients who were diagnosed with moderate-severe HIE and underwent hypothermia treatment during the period were included in the study. One hundred and eighteen of the patients were referred from other centers and only 7 (5.6%) patients were babies born in our hospital. During the follow-up, 68.8% (n = 86) of the patients were discharged from the hospital, and 31.2% (n = 39) of them died. Demographic characteristics (birth weight, sex, maternal age, gestational week, and delivery method) of both groups were statistically similar. When the exitus group was compared with the discharged group; we determined that the neonates who died had longer median resuscitation times in the delivery room [respectively, 10 (0–30) vs. 1 (0–20), p < 0.05], and lower 10^th^-min median APGAR scores [respectively, 4 (0–7) vs. 6 (3–10), p < 0.05] (Table 1). The distribution of asphyxia etiology of both groups was statistically similar (Table 2).

According to the laboratory results; exitus group versus discharged group; the median PH value [respectively, 6.87 (6.4–7.14) vs. 6.90 (6.58–7.12), p < 0.05], mean HCO_3_ value (respectively, 8.23 ± 2.67 vs. 10.33 ± 2.48, p < 0.05) and mean BE value [respectively, −22.39 ± (−4.42) vs. −19.46 ± (−3.95), p < 0.05] were lower than discharged group. Also, kidney (blood urea nitrogen and creatinine) and liver (aspartate aminotransferase [AST] and alanine aminotransferase [ALT]) function tests and values of potassium and phosphorus in the exitus group were higher. Levels of platelet and calcium were lower than discharged group (p < 0.05) (Table 3).

In the clinical observation of the exitus group, pulmonary hypertension progression and needs for peritoneal dialysis, surfactant, inotrope, and intubation were more frequent (p < 0.05). We determined that the frequency of seizure, abnormal aEEG, abnormal cranial ultrasonography findings were higher in the exitus group. We also determined that the first seizure time was earlier in the exitus group (p < 0.05) (Table 4).

We examined our patients in 2 groups as moderate and severe HIE. The mortality rate was statistically higher in the severe group (respectively, 86.1% vs. 9%, p < 0.001). In addition, liver and kidney dysfunction was more common in the group with severe HIE. Also, low pH and BE in the first blood gas were statistically significant in the severe group. The number of patients who had seizures was also higher in the severe HIE group (Table 5).

## 4. Discussion

Even though hypothermia is the only efficient proven treatment, HIE is still one of the significant causes of neonatal mortality. Because it may result in serious sequels for survivors, a better knowledge of prognostic factors about HIE is important for physicians who are treating patients. It would also allow physicians to inform patient relatives better about the neonate’s prognosis. The mortality rate is reported between 10%–60%, and approximately 24% of patients in neonatal intensive care units died before discharge. In survivors, motor and behavioral problems are seen as well as cerebral palsy and audio-visual problems [10–12]. According to the Turkish Neonatology Association 2008 data, the mortality rate of HIE-diagnosed patients is 22.6% [4]. Since our neonatal intensive care unit is the reference center in the region, 118 of the patients were referred from other centers and only 7 (5.6%) patients were babies born in our hospital. Passive cooling had been started in 76 of the patients that came to our unit from other centers. In our study group, we found the mortality rate of moderate-severe HIE diagnosed patients who underwent hypothermia treatment as 31.2% which is similar to literature data.

The neuroprotective efficiency of hypothermia treatment depends on the stage of HIE and the starting time of treatment. It is known that the mortality rate of Sarnat stage 3 HIE-diagnosed neonates is higher [13]. In our study group, the mortality rate of severe HIE patients was significantly higher than the moderate HIE group. The most effective period for hypothermia treatment is the period before the development of secondary energy deficiency [13]. Therefore, hypothermia should start in the postnatal first 6 hours. We started hypothermia treatment in postnatal first 6 h of all patients and the starting times of the two groups were statistically similar.

In neonates who are exposed to asphyxia, to protect the vital organs such as the heart and brain from hypoxia, blood flow reorganizes (diving reflex). This situation increases the damage rate of the organs which are directly affected by hypoxia such as the liver, kidney, and bowel. In their study, Havkins et al. [14] found that 80% liver damage, 72% kidney damage, 72% cardiac damage, and 54% hematological damage were accompanied by central nervous system damage to all patients who were exposed to asphyxia. They predict that low APGAR, low pH, and low BE in patients have the same predictive value as evidence of organ dysfunction. According to the study performed on 144 asphyxia neonates by Şah et al. [15], it was determined that at least one organ dysfunction appears except the nervous system. Alsina et al. [16] had evaluated 79 HIE patients in terms of renal, cardiovascular, respiratory, hepatic, hematological systems, and pH and electrolyte imbalance in their study. They found that pH and electrolyte imbalance and hepatic damage were most frequently seen. In their study, they determined that the severity of multi-organ dysfunction and the degree of hypoxia shows a positive correlation. In our study population, the rate of multi-organ dysfunction was higher in patients with severe HIE, following the literature.

HIE is the most important reason for neonatal convulsions and progress to convulsion is observed in the first 24 h in 50%–70% of neonates with HIE [17]. While convulsions are seen in the first hours for most of the cases, convulsions start right after the birth when it comes to severe HIE cases [18]. In addition to the frequency of seizures in the exitus group, we also determined that in our study the time of first seizures was earlier.

Liver damage mostly stems from hypoperfusion rather than hypoxia. According to the studies performed by Muniraman et al. [19], it was determined that transaminase levels (AST and ALT) increase in correlation with HIE grade. In their study, Sharma et al. [20] found that the severity of hepatic dysfunction correlates well with the increasing severity of asphyxia. Acute kidney damage is also one of the complications related to mortality and is most frequent among asphyxiating neonates. In their study Grossman et al. [21] found that, acute kidney damage incidence is 45% and is also related to neonatal mortality. In an 8-year retrospective study by Michnlewicz et al. [22], they found that AST, ALT, and creatinine levels were significantly higher and platelet levels were lower in the group with severe HIE. In their prospective cohort study with 150 infants with HIE, Acharya et al. [23] found that hypocalcemia, hyperkalemia, and high urea-creatinine levels were correlated with HIE grade. A higher incidence of multi-organ dysfunction was also observed in the exitus group and the severe HIE group in our study; similar to the literature. Elevation in transaminases and kidney function disruption rate was significantly higher in the exitus group and it was correlated with HIE grade. A study has shown that neonates with HIE who had thrombocytopenia had higher mortality [24]. Platelet values were statistically lower in the exitus group of our study. In a meta-analysis by Lakshminrusimha et al. [25], they found the pulmonary hypertension rate to be 22% in 303 infants with HIE and found that it was associated with mortality, a higher need for dialysis, surfactant and inotropic support, and a higher intubation rate. The rate of pulmonary hypertension in our patients was 21.6% and it was significantly higher in the exitus group.

In this study which we present our experience of 11 years, a deep acidosis in the first blood gas, abnormalities in laboratory values (electrolyte abnormality, liver, and kidney dysfunction) measured at 24^th^ h, early-onset seizures, and higher intubation rates were associated with a high risk of mortality. HIE is the 3^rd^ most common reason for death in the neonatal period. The risk of multi-organ failure development and sequel rate for the survivors is high in these patients. It is important to know and specify the poor prognostic factors early in this group that have high rates of mortality and forensic cases. This will help both to be prepared for the complications that may be encountered in the follow-up stage and intervene at early stages, and inform the parents in advance about the serious problems that may be encountered in the future.

## Figures and Tables

**Table 1:** Demographic characteristics and first evaluation of the patients who were exitus and discharged groups.

	All patients (n = 125)	Discharged groups (n = 86)	Exitus groups (n = 39)	p-value†
**Birth weight g** *	3175.0 ± 619.6	3194.6 ± 491.8	3131.7 ± 580.6	0.533
**Gestational weeks week** *	38.68 ± 1.89	38.66 ± 2	38.72 ± 1.5	0.881
**Gender (female) n (%)**	53 (42.4)	33 (38.3)	20 (51.2)	0.247
**Mode of delivery (Caesarean) n (%)**	58 (46.4)	39 (45.3)	19 (48.7)	0.876
**Maternal age years** *	29.38 ± 6.8	28.7 ± 6.7	30.87 ± 6.9	0.102
**Referred from other centers n (%)**	118 (94.4)	84 (97.67)	34 (87.17)	0.03
**Passive cooling n (%)**	76 (60.8)	48 (55.8)	28 (71.7)	0.134
**Active cooling starting time hours** *	2.40 ± 1.8	2.48 ± 1.9	0.72 ± 0.4	0.570
**First rectal temperature °C** **	35 (29–37)	35 (32–37.3)	34.5 (29–36.2)	**0.005**
**10th min APGAR score** **	5 (0–10)	6 (3–10)	4 (0–7)	**<0.05**
**Resuscitation time minutes** **	2 (0–30)	1 (0–20)	10 (0–30)	**<0.05**

* Values are given as mean ± standard deviation.

** Values are given as median (min-max).

† Results of statistical comparisons between discharged and exitus groups.

**Table 2:** Etiology distribution patients of hypoxic-ischemic encephalopathy.

	All patients (n = 125)	Discharged groups (n = 86)	Exitus groups (n = 39)	p-value†
**Meconium aspiration syndrome n (%)**	38 (30.4)	27 (31.4)	11 (28.2)	**0.269**
**Difficult delivery/Prolonged labor n (%)**	42 (33.6)	30 (34.9)	12 (30.8)	
**Cord entanglement n (%)**	9 (7.2)	7 (8.1)	2 (5.1)	
**Cord prolapse n (%)**	4 (3.2)	4 (4.6)	0	
**Placental abruption n (%)**	20 (16)	7 (8.1)	2 (5.1)	
**Fetal distress n (%)**	9 (7.2)	9 (10.5)	11 (28.2)	
**Chorioamnionitis n (%)**	2 (1.6)	1 (1.2)	1 (2.5)	
**Anemia n (%)**	1 (0.8)	1 (1.2)	0	

† Results of statistical comparisons between discharged and exitus groups.

**Table 3:** Blood gas values taken in the first hour and biochemical and complete blood count parameters taken in the 24^th^ h of patients.

	All patients (n = 125)	Discharged groups (n = 86)	Exitus groups (n = 39)	p-value†
**PH** **	6.89 (6.4–7.14)	6.90 (6.58–7.12)	6.87 (6.4–7.14)	**<0.05**
Base excess* **mmol/L**	−20.37 ± 4.30	−19.46 ± 3.95	−22.39 ± 4.42	**<0.05**
Bicarbonate* **mmol/L**	9.68 ± 2.72	10.33 ± 2.49	8.23 ± 2.67	**<0.05**
The partial pressure of carbon dioxide* **mmHg**	49.73 ± 24.93	47.67 ± 21.60	54.28 ± 30.90	0.176
White blood cell* **10^3/uL**	24.60 ± 9.94	23.48 ± 9.92	27.08 ± 9.65	0.06
Hemoglobin* **g/dL**	17.20 ± 5.85	17.27 ± 2.89	15.95 ± 3.64	0.109
Platelet* **10^3/uL**	209.90 ± 83.40	227.36 ± 78.88	171.41 ± 81.05	**<0.05**
Potassium* **mmol/L**	5.23 ± 1.19	5.07 ± 0.908	5.64 ±1.64	**0.016**
Calcium* **mg/dL**	8.02 ± 1.21	8.25 ± 0.84	7.67 ± 1.18	**0.003**
Phosphorus* **mg/dL**	6.17 ± 2.41	5.38 ± 1.33	8.11 ± 3.27	**<0.05**
Uric acid* **mg/dL**	9.36 ± 3.64	8.25 ± 2.72	12.17 ± 4.19	**<0.05**
Lactate dehydrogenase** **U/L**	1855 (611–11147)	1684 (611–11147)	3320 (706–6650)	**0.016**
Blood Urea Nitrogen* **mg/dL**	14.67 ± 6.37	13.48 ± 4.73	16.87 ± 8.88	**0.008**
Creatinine** **mg/dL**	0,97 (0.3–2.8)	0.80 (0.3–2)	1.07 (0.7–2.8)	**<0.05**
Aspartate aminotransferase** **U/L**	173 (31–4500)	135 (31–3607)	549 (45–4500)	**<0.05**
Alanine aminotransferase** **U/L**	55 (9–4200)	39 (9–1432)	267 (16–4200)	**<0.05**

* Values are given as mean ± standard deviation.

** Values are given as median (min-max).

† Results of statistical comparisons between discharged and exitus groups.

**Table 4:** The clinical follow-up information of patients

	All patients (n = 125)	Discharged groups (n =86)	Exitus groups (n = 39)	p-value†
**Seizures in the first 72 h n (%)**	69 (55.2)	37 (43)	32 (82.1)	**<0.05**
**First seizures time hour** *	4 (1–60)	6 (1–60)	2 (1–48)	**<0.05**
**Abnormal aEEG trace n (%)**	37 (29.6)	21 (24.4)	16 (41)	**0.001**
**Abnormal cranial USG n (%)**	65 (52)	30 (34.8)	35 (89.7)	**<0.05**
**Peritoneal dialysis n (%)**	15 (12)	2 (2.32)	13 (33.33)	**<0.05**
**Inotropic support n (%)**	68 (54.4)	32 (37.20)	36 (92.30)	**<0.05**
**Surfactant treatment n (%)**	25 (20)	10 (11.62)	15 (38.46)	**0.001**
**Pulmonary hypertension n (%)**	27 (21.6)	8 (9.30)	19 (48.71)	**<0.05**
**Intubation rate n (%)**	74 (59.2)	31 (36)	37 (94.8)	**<0.001**

* Values are given as median (min-max).

† Results of statistical comparisons between discharged and exitus groups.

**Table 5:** Evaluation of patients according to the grade of HIE.

	All patients (n = 125)	Moderate HIE (n = 89)	Severe HIE (n = 36)	p-value†
**Mortality rate n (%)**	39 (31.2)	8 (9)	31 (86.1)	**<0.001**
**PH** **	6.89 (6.4–7.14)	6.94 (6.6–7.12)	6.80 (6.4–7.14)	**<0.001**
Base excess* **mmol/L**	−20.37 ± 4.30	−19.23 ± 3.35	−23.23 ± 5.08	**<0.001**
Blood urea nitrogen* **mg/dL**	14.67 ± 6.37	13.65 ± 4.89	16.64 ± 8.96	0.077
Creatinine** **mg/dL**	0.97 (0.3–2.8)	0.8 (0.3–2.0)	1.07 (0.6–2.8)	**0.001**
Aspartate aminotransferase** **U/L**	173 (31–4500)	136.5 (31–3607)	549 (32–4500)	**<0.001**
Alanine aminotransferase** **U/L**	55 (9–4200)	40 (9–1432)	267 (23–4200)	**<0.001**
Platelet* **10^3/uL**	209.90 ± 83.40	220.34 ± 82.86	184.08 ± 80.11	**0.027**
**Seizures in the first 72 h n (%)**	69 (55.2)	37 (41.57)	32 (88.8)	**<0.001**

* Values are given as mean ± standard deviation.

** Values are given as median (min-max).

† Results of statistical comparisons between moderate and severe HIE groups.

## Data Availability

The datasets used and/or analyzed during the current study are available from the corresponding author on reasonable request.
